# Antimicrobial and Mechanical Properties of Orthodontic Acrylic Resin Containing Zinc Oxide and Titanium Dioxide Nanoparticles Supported on 4A Zeolite

**DOI:** 10.1155/2022/8155971

**Published:** 2022-07-14

**Authors:** Mahdiyeh Esmaeilzadeh, Baharak Divband, Bahram Ranjkesh, Fatemeh Pournaghi Azar, Fatemeh Yeganeh Sefidan, Mojgan Kachoei, Behnaz Karimzadeh

**Affiliations:** ^1^Dental and Periodontal Research Center, Tabriz University of Medical Sciences, Tabriz, Iran; ^2^Department of Dentistry and Oral Health, Section for Prosthetic Dentistry, Aarhus University, Vennelyst Boulevard 9, 8000 Aarhus C, Aarhus, Denmark; ^3^Department of Operative Dentistry, Faculty of Dentistry, Tabriz University of Medical Sciences, Tabriz, Iran; ^4^Department of Bacteriology and Virology, Faculty of Medicine, Tabriz University of Medical Sciences, Tabriz, Iran; ^5^Department of Orthodontics, Faculty of Dentistry, Tabriz University of Medical Sciences, Tabriz, Iran; ^6^Student Research Committee, Faculty of Dentistry, Tabriz University of Medical Sciences, Tabriz, Iran

## Abstract

Polymethyl methacrylate (PMMA) is widely used to manufacture removable orthodontic appliances. However, since the porous structure, cold-curing acrylic resins are susceptible to bacterial adhesion and colonization. The aim of this study was to investigate the antibacterial and mechanical properties of a cold-curing PMMA resin containing ZnO and TiO_2_ nanoparticles supported on the 4A zeolite. ZnO and TiO_2_ nanoparticles supported on the 4A zeolite were synthesized. Nanoparticles were added in three compositions as ZnO/4A, TiO_2_/4A, and ZnO/TiO_2_/4A at 2wt% and 4wt% concentrations to cold-curing acrylic resin powder (SR Triplex® Cold Ivoclar Vivadent AG, FL-9494 Schaan/Liechtenstein). X-ray diffraction (XRD), Field Emission Scanning Electron Microscopy (FE-SEM), energy dispersive X-ray (EDX), transmission electron microscopy (TEM), and dynamic light scattering (DLS) were performed to investigate the nanocomposite characteristics. A direct test method was used to assess the antibacterial properties against *Streptococcus mutans, Klebsiella pneumoniae,* and *Escherichia coli*. The surface roughness of acrylic samples was measured with a profilometer. Flexural strength was evaluated by a three-point bending test, and one-way ANOVA and Tukey's post hoc tests were used for statistical evaluation of the data. A *p* value of less than 0.05 was considered statistically significant. XRD confirmed the accurate crystalline structure of synthesized nanoparticles; FE-SEM images showed nanoparticle dispersion within polymerized acryl. The addition of 2 and 4 wt% of ZnO/4A, TiO_2_/4A, and ZnO/TiO_2_/4A caused colony reduction in all types of tested microorganisms more than 99% and 100%, respectively. The mean flexural strengths of acrylic specimens containing 2wt% and 4wt% of synthesized nanoparticles were significantly lower than those of the resin without nanoparticles. Fabricated samples showed favorable antibacterial properties but decreased flexural strength.

## 1. Introduction

Removable orthodontic appliances are indicated as a retainer after fixed orthodontic treatments to slightly move the teeth and in functional treatments, as well [1]. Despite advantages, the accumulation of microbial plaque on dental surfaces and areas of retaining components, mostly on acrylic base plates, stands as their disadvantage [1]. Cold-curing acrylic resins, mainly composed of poly (methyl methacrylate (PMMA)), are widely used to fabricate removable orthodontic appliances [1, 2], due to its desirable properties such as acceptable mechanical properties, biocompatibility, availability, cost-effectiveness, easy handling, and optimal esthetic [3, 4]. The polymerization of polymethyl methacrylate causes porosity in its bulk structure. The overall porosity depends on the monomer's degree of conversion to the polymer [1, 5, 6]. Studies have shown that cold-curing acrylic resins have more porosity than heat-curing acrylic resins because of the low degree of polymerization in cold-curing acrylics which would eventually lead to higher water absorption and increased risk of bacterial adhesion and plaque colonization. Microorganisms such as *Streptococcus mutans and Candida albicans* can penetrate 1 to 2 nm into the acrylic baseplate surface [7, 8]. The microbial activities of these microorganisms could cause malodor production stemming from orthodontic base plates, which has negatively influenced the patients' cooperation [8]. Further, removable orthodontic appliances can change the mouth's microbial flora and increase the rate of *Streptococcus mutans* which increases the tendency to develop dental caries [9]. Therefore, in orthodontic patients, the use of antimicrobial chemicals such as chlorhexidine gluconate mouthwash is recommended because mechanical brushing is not capable of optimal eradicating microorganisms from all plaque retentive areas. Chlorhexidine mouthwash is the gold standard compared to other antimicrobial chemicals, though it is not satisfactory in many patients due to its side effects, such as taste changes and discoloration of the teeth [8]. Taking it all together, doping the acrylic base plate structure with appropriate additives that may improve the antimicrobial properties of the appliance without hampering its mechanical properties would be desirable. Farhadian et al. exhibited that silver nanoparticles addition with a size of 40 nm at 500 ppm concentration to the acrylic base plate significantly reduced the colony formation of *Streptococcus* mutans [9]. Ghaffari and Hamedi-rad show that the addition of silver nanoparticles with a concentration of 5wt% to cold-curing PMMA acrylic resin reduces its tensile strength [10]. Studies have shown the antimicrobial properties of zinc oxide and titanium dioxide nanoparticles on Gram-positive, Gram-negative, and fungal infections. In addition, their biocompatibility and nontoxicity have led to their widespread use in various polymers and materials [3, 11–13]. Ceirech et al. incorporated zinc oxide nanoparticles into heat-curing PMMA which could reduce the *Candida albicans* accumulation and decrease the incidence of denture stomatitis [14] with no adverse effects on the mechanical properties of the denture [15]. Antibacterial zeolites have been investigated extensively recently. Zeolites are aluminosilicate particles with a crystalline structure with 3–10 angstrom voids in their structure. These spaces are suitable for exchanging antimicrobial cations, such as Ag, Zn, and Ti [6]. Today, with the widespread use of zeolite synthesis, they are used as supports to improve the performance of metal oxide nanoparticles that have antimicrobial properties [16]. The aim of this study was to investigate the antibacterial and mechanical properties of cold-curing PMMA acrylic resin containing ZnO and TiO_2_ nanoparticles supported in the 4A zeolite framework at three different compositions (only ZnO/4A, only TiO_2_/4A, and the combination of ZnO and TiO_2_/4A) at 2wt% and 4wt% concentrations.

## 2. Materials and Methods

The present study has been performed experimentally and *in vitro*. All procedures have been approved by the ethics committee of Tabriz University of Medical Sciences with ethical number IR.TBZMED.VCR.REC.1398.350.

### 2.1. 4A Zeolite Synthesis

Hydrothermal synthesis of 4A zeolite particles with silica to aluminum ratio of SiO_2_/Al_2_O_3_ ≅ 2 was performed [17]. In this process, alumina and clinoptilolite were used as sources of aluminum and silica and mixed with NaOH. The final solution was transferred to Teflon containers and then subjected to a constant temperature and pressure to form the ultimate 4A zeolite network by crystallization. The next step was to remove the synthesized zeolite precipitate from Teflon containers and wash it several times to reduce the pH and other unreacted materials. Then, the white powder of 4A zeolite was dried overnight at 100°C. The ion exchange method was used to embed ZnO and TiO_2_ nanoparticles on the as-synthesized 4A zeolite.

### 2.2. 4A Zeolite Containing Zinc Oxide Nanoparticles (ZnO/4A) Synthesis

A solution of 0.17 g Zn (CH_3_CHOO)_2_·2H_2_O was prepared and added to the zeolite suspension at a ratio of 5 : 95 (wt.%) and mixed for 30 minutes. The ion exchange process was performed at 60°C for 24 hours; finally, the particles were separated by centrifuge, washed to remove extra Zn ions, and dried at room temperature, and calcination was performed at 550°C.

### 2.3. 4A Zeolite Containing Titanium Dioxide Nanoparticles (TiO_2_/4A) Synthesis

A solution with a particular concentration of ortho-titanate (0.2 g) was dissolved in 2 mL ethanol 96% and added to the zeolite 4A suspension at a ratio of 5 : 95 (wt/wt%) and mixed for 30 minutes. The ion exchange process was performed at 90°C for 6 hours. Finally, the particles were separated by centrifuge, washed, and dried at room temperature, and calcination was performed at 550°C.

### 2.4. 4A Zeolite Containing Zinc Oxide and Titanium Dioxide Nanoparticles (ZnO/TiO_2_/4A)

A suspension of zinc oxide nanoparticles and zeolite 4A with a ratio of 2.5 : 95 (wt%/wt%) was prepared. Then, titanium dioxide powder with a 2.5 : 95 (wt%/wt%) ratio was added to the suspension. Finally, after ion exchange, the produced particles were separated by centrifuging and dried at room temperature. Then, it was calcined in a furnace at 550°C, and zeolite 4A nanoparticles containing zinc oxide and titanium oxide nanoparticles were synthesized.

### 2.5. Specimen Preparation and Structural Characterization

The as-synthesized ZnO/4A, TiO_2_/4A, and ZnO/TiO_2_/4A nanoparticle powder with 2wt% and 4wt% concentrations was added to SR Triplex® Cold (Ivoclar Vivadent AG, FL-9494 Schaan/Liechtenstein) acrylic powder. After mixing manually, the combined powder was shaken in an amalgamator for 10 minutes to ensure a homogeneous and uniform mixture. Finally, 6 test groups and a control group were prepared (Table 1). According to the manufacturer's instruction, 10 mL liquid monomer was manually mixed with 13 g of powder.

#### 2.5.1. X-Ray Diffraction (XRD)

It was performed by Bruker D5000 X-ray diffraction system (Siemens, Germany) at room temperature, at Cu k*α* radiation (*λ* = 1.5418 A and 2*θ* = 4–70°), 40 kV, and 30 mA to investigate the crystalline structure of the samples.

#### 2.5.2. Field Emission Scanning Electron Microscopy (FE-SEM)

Field Emission Scanning Electron Microscope (TESCAN, Brno, Czech Republic) equipped with energy dispersive X-ray (EDX) was used to study morphology and microstructure analysis of the acrylic resin containing synthesized nanoparticles. X-ray scattering spectroscopy was used for structural and elemental analysis of the samples. SEM samples were gold-coated before the scanning. SEM was performed at 5 kV and EDX was done at 15 kV. MAP analysis was performed using the wavelength-dispersive X-ray spectroscopy system (WDS) to analyze uniform and homogeneous nanoparticle and element distribution.

#### 2.5.3. Transmission Electron Microscopy

Transmission electron microscopy (Philips CM120, Eindhoven, The Netherlands) was operated at 100 kV to investigate the exact size of synthesized nanoparticles and obtain the particle size distribution.

#### 2.5.4. Dynamic Light Scattering (DLS)

Dynamic light scattering (Zetasizer Nano ZS ZEN 3600, Malvern Instruments Ltd, UK), tuning to a wavelength of 633 nm, was used for nanoparticle size measurement, dispersion, and the zeta potential assessment. Distilled water was used as the suspension media of nanoparticles during the evaluation. Particle size was determined by the ID (intensity distribution) index, and polydispersity index (PDI) was used to evaluate the particle dispersion.

#### 2.5.5. Surface Roughness

The surface roughness of acrylic samples was measured using Sharif solar profiling device (PFM-3320, Iran). A sample with dimensions of 10 × 10 mm and thickness of 1.2 ± 0.2 mm was prepared from each group (*n* = 7). For the purpose of surface standardization, all specimens were polished with 800-grit silicon and 1200-grit carbide abrasive papers for 60 seconds. Afterward, all seven samples were cleaned with air pressure and examined in the profilometer. A steel needle tip measured the surface roughness passed through the surface of the specimens at a speed of 0.2 mm/s with a constant force, and finally, the average values of surface roughness were determined for each group.

### 2.6. Antibacterial Characterization of Samples

Six-well cell culture plates were used for the antibacterial test. 10 mL of monomer was poured into each of the wells according to the manufacturer's instruction, and 13 g of PMMA powder containing nanoparticles was added to the monomer by sprinkle-on technique. All samples were stored at room temperature for six minutes for initial polymerization. The positive control group was PMMA without any additive nanoparticle. The negative control was acrylic resin containing silver nanoparticles and its antimicrobial properties have been proven [18].

Three bacterial strains of *Escherichia coli* (ATCC 25922), *Klebsiella pneumoniae* (ATCC 700603), and *Streptococcus mutans* (PTCC 1683) were used for antimicrobial testing. Bacterial strains were cultured separately under the sterile condition on BHI (Brain Heart Infusion agar, Merck, Darmstadt, Germany) agar at 37°C for 24 hours. After the incubation period, the standard 0.5 McFarland concentration of each bacteria (containing approximately 1.5 × 10^8^CFU/mL) was prepared into the BHI broth. Under the sterile condition, 1 mL of the prepared concentration was inoculated to each of the wells and incubated at 37°C for 24 hours. After the incubation period, approximately 20 *μ*L from each well was transferred to an agar medium to study microorganisms' growth. For each well that showed growth, several dilutions were prepared to count the number of bacterial colonies. From each dilution, 100 *μ*L was inoculated into a standard agar and culture with the spread plate method and incubated at 37°C for 24 hours. After the incubation, the number of colonies was counted, and the number of colonies was reported in CFU/mL. These steps were repeated for each bacterial strain.

### 2.7. Flexural Strength

To evaluate the flexural strength of the acrylic samples, the three-point bending test was performed by a universal testing machine (Hounsfield H5K–S, England). The crosshead was calibrated at a speed of 5 mm/min, and the distance between the supports was adjusted to 50 mm. Five specimens from each group (two test groups and one control group) were prepared in the form of strips with a length of 64 mm and a width of 10 ± 0.2 mm and a thickness of 3.3 ± 0.2 mm. The samples were made according to the ISO standard 20795-2:2013. The maximum force was recorded in Newton and then applied to the following formula (1), where *F* is the maximum load, *l* is the distance between the supports of the device, *b* is the width of the samples, and *h* is the height of the samples.

(1) $$\begin{array}{c} \;\text{Flexural strength }=3FL/2bh^{2}. \end{array}$$

The ultimate flexural strength was reported in Mega Pascal (MPa). One-way ANOVA and Tukey's post hoc multiple comparisons test were used for statistical analysis at a significance level of 0.05.

## 3. Results

**Figure 1:**
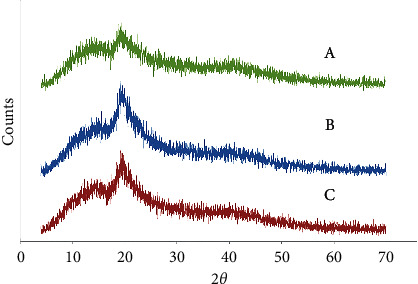
The X-ray diffraction pattern related to crystalline structures within the polymerized acrylic resin containing (a) ZnO/4A, (b) TiO_2_/4A, and (c) ZnO/TiO_2_/4A.

**Figure 2:**
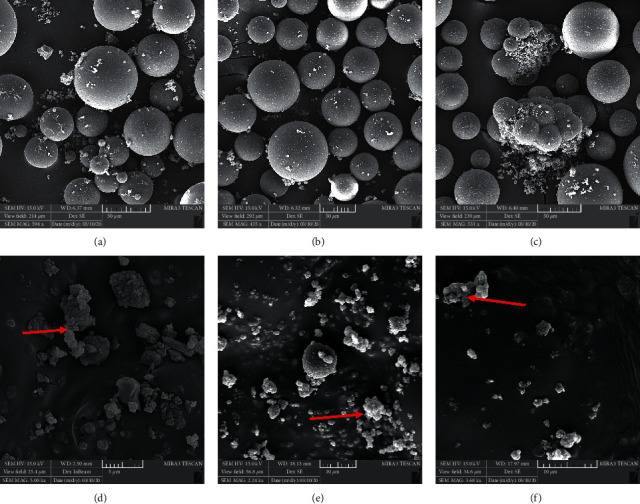
FE-SEM images of acrylic powder containing (a) ZnO/TiO_2_/4A, (b) TiO_2_/4A, and (c) ZnO/4A. FE-SEM images of polymerized acryl containing (d) ZnO/TiO_2_/4A, (e) TiO_2_/4A, and (f) ZnO/4A. Accumulation of 4A zeolites observed in some areas indicated by red arrows.

**Figure 3:**
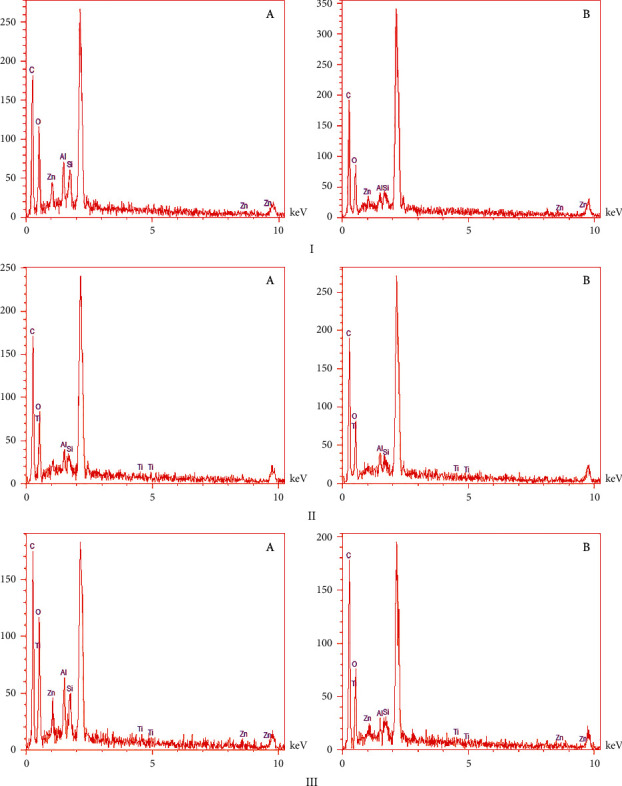
EDX analysis of polymerized acrylic resin containing I (ZnO/4A, II) (TiO_2_/4A, III) ZnO/TiO_2_/4A ZA. (a) stands for 4wt% and (b) for 2wt% of each nanoparticle.

**Figure 4:**
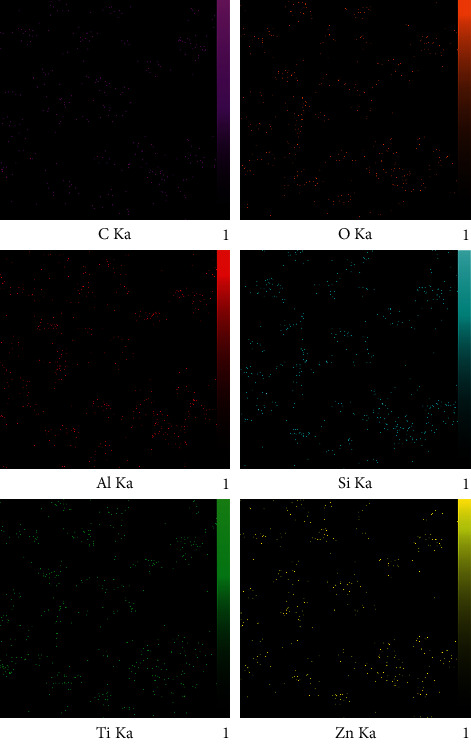
MAP image of polymerized acryl containing ZnO/TiO_2_/4A nanoparticles.

**Figure 5:**
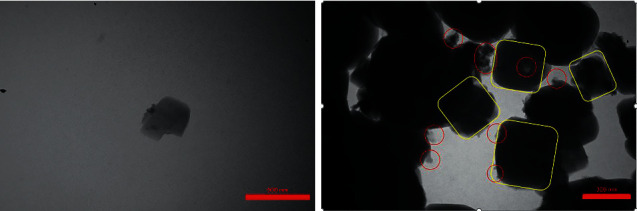
TEM image of ZnO/TiO_2_/4A nanoparticles: yellow cubes represent 4A zeolite and red circles represent zinc and titanium oxide nanoparticles.

**Figure 6:**
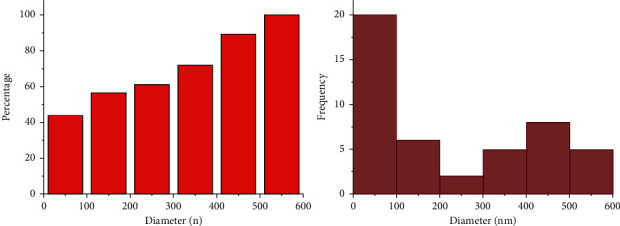
Histogram of synthesized ZnO/TiO_2_/4A nanoparticle size distribution.

**Figure 7:**
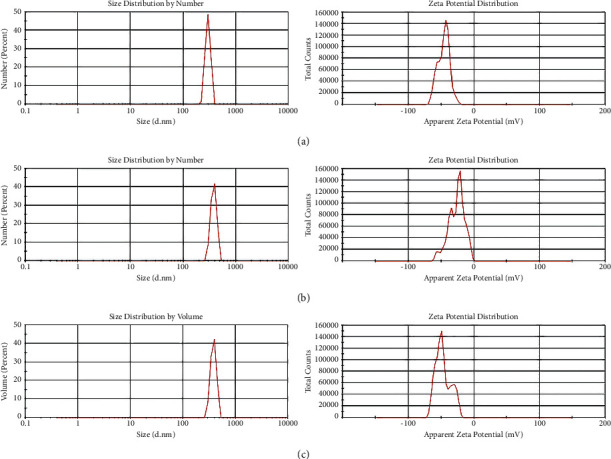
DLS analysis and zeta potential of (a) ZnO/4A, (b) TiO_2_/4A, and (c) ZnO/TiO_2_/4A nanoparticles.

1. The X-ray diffraction pattern is related to crystalline structures within the polymerized acrylic resin containing ZnO/4A, TiO_2_/4A, and ZnO/TiO_2_/4A nanoparticle powder (Figure 1). Since the polymer powder is mixed with nanoparticles, the amorphous structure of the polymer creates a hump-like shape in the XRD pattern.
2. FE-SEM images of acrylic powder containing different nanoparticle powders indicate the adherence or dispersion of synthesized nanoparticles between the acrylic particles (Figure 2). The proper distribution of ZnO/4A, TiO_2_/4A, and ZnO/TiO_2_/4A ZA nanoparticles is evident in the polymerized acryl images (Figure 2); nevertheless, the accumulation of nanoparticles is observed in some areas.
3. The results of total elemental and structural analysis (EDX) analysis of polymerized acrylic resin containing ZnO/4A, TiO_2_/4A, and ZnO/TiO_2_/4A nanoparticles with two different concentrations of 2wt% and 4wt% showed the presence of silicon, aluminum, oxygen, zinc, or/and titanium atoms, confirming the presence of zeolite and zinc oxide or/and titanium dioxide in acrylic resin (Figure 3). Moreover, in the EDX-mapping images, the elements in all samples showed an almost uniform and homogeneous distribution (Figure 4).
4. The synthesized quadrilateral zeolites and metal oxide nanoparticles with smaller sizes can be detected based on transmission electron microscopy (TEM) images (Figure 5). The particle size histogram is consistent with FE-SEM images. In the particle dispersion histogram, particles smaller than 100 nm indicate the presence of zinc oxide-titanium oxide nanoparticles and particles larger than 100 nm are related to zeolite particles (Figure 6).
5. Dynamic light scattering results of nanoparticle powder suspension in distilled water are shown in Figure 7. According to the particle size distribution and dispersion diagram, the nanoparticle size profile shows an average of 295 nm for ZnO/4A nanoparticles, an average of 380 nm, and 381 nm for TiO_2_/4A and ZnO/TiO_2_/4A, respectively. High zeta potential indicates the higher stability of particles in an aqueous solution. The surface charge of nanoparticles based on zeta potential for ZnO/4A, TiO_2_/4A, and ZnO/TiO_2_/4A is −45.9, −26.2 mV, and −47 mV, respectively.
6. Based on profilometry evaluations, the surface roughness for the control group is 0.4 *μ*m. Among the 6 test groups, the highest surface roughness was related to the G4 group (acrylic resin containing ZnO/TiO_2_/4A 4wt%) and was 0.6 *μ*m, whereas an acrylic resin containing the same nanoparticle with a concentration of 2wt% showed even more minor surface roughness than the control group, and 0.3 *μ*m was recorded. G2 and G6 groups (acrylic resin containing ZnO/4A 2wt% and 4wt% concentrations) showed a relatively similar surface roughness to the G1 group and less than the control group which is equal to 0.3 *μ*m. The G3 and G5 groups (acrylic resin containing 2wt% and 4wt% TiO_2_/4A) were reported to be 0.5 and 0.4 *μ*m, respectively.

### 3.1. Antimicrobial Assessment

The results of bacterial colony count are given in Table 2. The 4wt% of three types of nanoparticles and concentration of 2wt% of TiO_2_/ZnO/4A leads to 100% elimination of colonies in three bacterial strains. In the case of ZnO/4A and TiO_2_/4A with a concentration of 2wt%, only a small number of *Klebsiella pneumoniae* colonies were formed, which is significantly lower than the control sample.

### 3.2. Flexural Strength

The results of the flexural strength are given in Table 3. The mean flexural strength in acrylic specimens containing ZnO/TiO_2_/4A, TiO_2_/4A, and ZnO/4A is significantly lower than that of the control group (*P* < 0.05). It is noteworthy that after the control group (85.85 MPa), the highest mean flexural strength among the test groups belonged to the G1 group, which is equal to 71.90 MPa. This difference in the flexural strength of the G1 group compared to other test groups is significant (*P* < 0.05) except for the G6 group, where despite the higher flexural strength, this difference is not significant (*P*=0.305). According to the values in Table 3, the concentration of 2wt% of ZnO/TiO_2_/4A nanoparticles is significantly lower than the 4wt% concentration of this nanoparticle (*P*=0.003). Despite the considerable reduction in flexural strength of test groups, all mean values are greater than 50 MPa.

## 4. Discussion and Conclusion

The present study evaluated the incorporation of zinc oxide and titanium dioxide nanoparticles supported on the 4A zeolite to cold-curing PMMA and investigated the antibacterial and mechanical properties following characterization. Orthodontic treatments with the diagnosis and treatment of maxillofacial abnormalities play an essential role in creating the esthetic, function, and morphology of the craniofacial area in children and adults [19, 20]. Removable orthodontic appliances are widely used to treat dental and skeletal problems in the ages of 6 to 12 years, which coincides with the mixed dental ages. These appliances are easily inserted and removed by the patient and the dentist; further, they are made in the laboratory on dental casts. Removable appliances must be kept in the mouth for the majority of the day and night and should only be removed for eating and cleaning the mouth and appliance [20]. Self-curing poly (methylmethacrylate) acrylic resin is a prevalent material for manufacturing removable orthodontic base plates. After polymerization and hardening, this material acquires a porous structure due to evaporation of the residual monomer that has not entered the polymerization process. A variety of bacteria are present in the microbial flora of the oral cavity and can colonize on different surfaces. The acrylic base plate of orthodontic appliances is also a suitable place for the growth of various oral bacteria, despite the finishing and polishing processes; due to its uneven structure and very small porosities, accumulation of food debris along with colonization of microorganisms on acrylic base plate leads to metabolic activities of microorganisms. Volatile sulfide compounds (VSCs), such as hydrogen sulfide (H_2_S) and methyl mercaptan, are by-products of the metabolic activity of proteolytic, anaerobic, and Gram-negative bacteria [21]. Removable orthodontic appliances are mainly used for functional developmental treatments in preadolescent patients. Thus, they must be placed in the patient's mouth for long hours; moreover, the desired treatment outcomes are highly dependent on patient cooperation. In many cases, a lack of desire and motivation to use the removable appliance is the unpleasant odor of volatile sulfide compounds (VSCs) produced after several months of using the appliance, disrupting the patient's cooperation and the treatment failure [1, 8, 9]. In this study, we tested three bacterial strains: *Escherichia coli*, *Klebsiella pneumonia*, and *Streptococcus mutans*. Previous studies performed on denture bases typically made of heat-curing polymethyl methacrylate have shown that the most colonized microorganisms on acrylic bases are *Klebsiella pneumonia* and *Escherichia coli* [22]. In the population of denture users, bad breath is due to the metabolic reactions of the proliferation of Gram-negative bacteria, including the Enterobacteriaceae family [23]. The use of orthodontic appliances also leads to increased colonization of *Streptococcus* mutans, which has been indicated in various studies as the main cause of dental caries [1, 8]. In a study conducted by Farhadian et al. [9], as a clinical trial, silver nanoparticles were added to the acrylic base plate of orthodontic retainers, and their effect on the formation of *Streptococcus mutans* colonies was measured. It has been concluded that the addition of these nanoparticles has significantly reduced the colonies of this bacterium. In this study, the addition of ZnO/TiO_2_/4A, TiO_2_/4A, and ZnO/4A nanoparticles in both concentrations of 2wt% and 4wt% prevented the growth of three strains of bacteria: *Escherichia coli*, *Klebsiella pneumonia*, and *Streptococcus mutans*. Farhadian study's limitation was the lack of evaluation of adding silver nanoparticles to the acrylic base plate on its mechanical properties. In this study, we evaluated the flexural strength of acrylic samples containing ZnO/TiO_2_/4A, TiO_2_/4A, and ZnO/4A nanoparticles. In previous studies, the addition of silver nanoparticles to self-curing or heat-curing PMMA acrylic resins has improved antimicrobial properties [6, 10, 16]. However, the biosafety of silver nanoparticles and the risk of tooth discoloration (pigmentation) impair their clinical use, and silver nanoparticles should be used in limited size and concentration, which is a bit of a challenge; as a result, studies have recommended applying safer nanoparticles such as titanium dioxide and zinc oxide or copper oxide. The antimicrobial properties of zinc oxide nanoparticles have been proven in several studies [18, 24, 25]. Studies have also shown that ZnO nanoparticles up to 100 ppm do not have significant toxicity to human cells and are used in many industries [18]. Therefore, the choice of these nanoparticles to improve the antimicrobial properties of dental materials seems relevant. Using titanium dioxide nanoparticles in the food industry has been proven, and as a result, they are not toxic to humans and are safe nanoparticles with antimicrobial properties [17, 18]. One of the new methods for incorporating metal oxide nanoparticles into polymeric materials is to use a supporter with larger dimensions and a porous matrix with the aim of reducing their aggregation and leaching from the surface or inside of industrial compounds or dental materials; various synthesis procedures have been used; one of these is incorporating metal oxide nanoparticles in the porous matrix of zeolites which leads to enhancing antimicrobial properties [17, 26]. Zeolites are three-dimensional porous aluminosilicate nanostructures composed of SiO_4_ and AlO_4_ tetrahedrons joined by oxygen atoms to form a crystal structure with cavities and channels of atomic size. This unique structure allows cations to come into direct contact with microorganisms [26]. The antimicrobial properties of zeolites integrated with these antimicrobial cations in dental materials have been proven [6, 16]. These recombinant materials are nontoxic and biocompatible, have long-term antimicrobial properties, and have no taste [26]. Zeolite A, which is used in the present study, is a synthetic zeolite used as an adsorbent in various industries. The adsorption and desorption of H_2_S (hydrogen sulfide) have been investigated on two synthetic zeolites, zeolite A and ZSM-5. According to the results, the adsorption rate of hydrogen sulfide by zeolite A is higher than zeolite ZSM-5 [27]. The absorption and desorption of H_2_S from zeolite materials are due to the electrostatic interaction between H2S and absorbent [27]. These favorable properties have led to the widespread use of zeolites, and in the present study, zinc oxide and/or titanium oxide nanoparticles were supported by 4A zeolite and added to polymethyl methacrylate polymer. Based on a study that investigated the surface charge of polymer nanoparticles, it was found that the negative zeta potential of nanoparticles is less toxic and more biocompatible than their positive potential. However, in terms of nanoparticle stability, the higher the surface charge of nanoparticles, the better their stability values in the range of ±40 to ±60 mV for nanoparticles provide satisfying stability [28]. Synthesized ZnO/TiO_2_/4A and ZnO/4A nanoparticles with a zeta potential of −47 and −45.9 mV respectively have good stability and high biocompatibility but TiO_2_/4A nanoparticles with a zeta potential of −26.2 mV are less stable. Among the mechanical properties of acrylic resins, flexural strength is of great importance, and a minimum standard for all dental polymers, including acrylic resins, has been set by the World Standards Organization ISO 20795 (2008). Casemiro et al. [6] investigated the effect of adding silver-zinc zeolite nanoparticles on two types of heat-curing acrylic and a microwave-polymerized acrylic used to make dentures for antimicrobial and flexural strength. This study showed that the addition of more than 2.5% zeolite to QC20 and Lucitone 550 heat-curing acrylic resins and microwave-polymerized acrylic resin (Onda-Cryl) significantly reduced the flexural strength compared to the control group. However, in concentrations of less than 5%, all values obtained from all groups' flexural strength complied with ISO 1567 and were more than 65 MPa. The results of our study also showed that the addition of ZnO/TiO_2_/4A, TiO_2_/4A, and ZnO/4A nanoparticles to orthodontic self-curing acrylic resin in both 2 and 4wt% concentrations significantly reduces the flexural strength but complies with ISO number 20795-2: 2013, and all values are above 50 MPa. The orthodontic base plate has a lower ultimate flexural strength than a denture because it is removed from the mouth during feeding, whereas the denture stays in place. Antibacterial characteristics of orthodontic base plates were improved by the addition of nanoparticles of ZnO/TiO_2_/4A, TiO_2_/4A, and ZnO/4A. To find out if the antibacterial property of these baseplates affects bad breath, unpleasant odor of baseplates, tooth decay risk, patient compliance, and treatment effectiveness, clinical trials should be conducted in future studies.

## Figures and Tables

**Table 1:** Classification of test and control groups based on the type and percentage of nanoparticles added to the composition of SR Triplex® cold powder.

Group no.	Type of nanoparticle	wt%
G1	ZnO/TiO_2_/4A	2
G2	ZnO/4A	2
G3	TiO_2_/4A	2
G4	ZnO/TiO_2_/4A	4
G5	TiO_2_/4A	4
G6	ZnO/4A	4
G7	Without nanoparticle	0

**Table 2:** Initial concentration and concentrations of the three bacterial strains in suspensions after 24 h of contact with differently modified samples (colony formed unit/mL).

		Bacteria strain		
		*E. coli*	*S. mutans*	*K. pnemoneai*
Initial concentration (CFU/mL)		1.5 × 10^8^CFU/ml	1.5 × 10^8^CFU/ml	1.5 × 10^8^CFU/ml
Concentration after 24 h (CFU/mL)	ZnO/TiO_2_/4A 2% (**G1**)	0	0	0
	ZnO/4A2% (**G2**)	0	0	9 × 10^7^CFU/ml
	TiO_2_/4A (**G3**)	0	0	1 × 10^9^CFU/ml
	ZnO/TiO_2_/4A4% (**G4**)	0	0	0
	TiO_2_/4A4 (**G5**)	0	0	0
	ZnO/4A4% (**G6**)	0	0	0
	Control (**G7**)	3.4 × 10^10^CFU/ml	4.7 × 10^10^CFU/ml	2.4 × 10^10^CFU/ml

**Table 3:** Descriptive values of flexural strength (MPa).

Group	*N*	Mean	Std. deviation	Std. error	95% confidence interval for mean	
					Lower bound	Upper bound
TiO_2_/ZnO/4A 2% (G1)	5	60.0140	3.07822	1.37662	56.1919	63.8361
ZnO/4A 2% (G2)	5	62.6440	4.41532	1.97459	57.1617	68.1263
TiO_2_/4A 2% (G3)	5	56.1320	2.90165	1.29766	52.5291	59.7349
TiO_2_/ZnO/4A 4% (G4)	5	71.9000	1.83154	.81909	69.6258	74.1742
TiO_2_/4A 4% (G5)	5	61.2380	3.77233	1.68704	56.5540	65.9220
ZnO/4A 4% (G6)	5	65.7460	7.44352	3.32884	56.5037	74.9883
Control (G7)	5	85.8560	4.58846	2.05202	80.1587	91.5533
Total	35	66.2186	10.15395	1.71633	62.7306	69.7066

## Data Availability

The data that support the findings of this study are available from the corresponding author upon request.
